# Time-Dependent Propensity Score for Assessing the Effect of Vaccine Exposure on Pregnancy Outcomes through Pregnancy Exposure Cohort Studies

**DOI:** 10.3390/ijerph110303074

**Published:** 2014-03-12

**Authors:** Ronghui Xu, Yunjun Luo, Robert Glynn, Diana Johnson, Kenneth L. Jones, Christina Chambers

**Affiliations:** 1 Department of Family and Preventive Medicine, University of California, San Diego, 9500 Gilman Drive, MC 0112, La Jolla, CA 92093, USA; E-Mail: chchambers@ucsd.edu; 2 Department of Mathematics, University of California, San Diego, 9500 Gilman Drive, MC 0112, La Jolla, CA 92093, USA; 3 Department of Pediatrics, University of California, San Diego, 9500 Gilman Drive, La Jolla, CA 92093, USA; E-Mails: yuluo@ucsd.edu (Y.L.); d4johnson@ucsd.edu (D.J.); klyons@ucsd.edu (K.L.J.); 4 Department of Medicine, Brigham & Women's Hospital and Harvard Medical School, 1620 Tremont Street, Boston, MA 02120, USA; E-Mail: rglynn@partners.org

**Keywords:** Cox regression, pH1N1 influenza vaccine, preterm delivery

## Abstract

Women are advised to be vaccinated for influenza during pregnancy and may receive vaccine at any time during their pregnancy. In observational studies evaluating vaccine safety in pregnancy, to account for such time-varying vaccine exposure, a time-dependent predictor can be used in a proportional hazards model setting for outcomes such as spontaneous abortion or preterm delivery. Also, due to the observational nature of pregnancy exposure cohort studies and relatively low event rates, propensity score (PS) methods are often used to adjust for potential confounders. Using Monte Carlo simulation experiments, we compare two different ways to model the PS for vaccine exposure: (1) logistic regression treating the exposure status as binary yes or no; (2) Cox regression treating time to exposure as time-to-event. Coverage probability of the nominal 95% confidence interval for the exposure effect is used as the main measure of performance. The performance of the logistic regression PS depends largely on how the exposure data is generated. In contrast, the Cox regression PS consistently performs well across the different data generating mechanisms that we have considered. In addition, the Cox regression PS allows adjusting for potential time-varying confounders such as season of the year or exposure to additional vaccines. The application of the Cox regression PS is illustrated using data from a recent study of the safety of pandemic H1N1 influenza vaccine during pregnancy.

## 1. Introduction

Routine annual seasonal influenza vaccination is recommended for all persons six months of age or older. In particular, women are advised to be vaccinated for influenza during pregnancy and may receive vaccine at any time during their pregnancy [1]. In observational studies of vaccine safety in pregnancy, for certain pregnancy outcomes such as spontaneous abortion or preterm delivery, the fact that vaccination can happen at any time during pregnancy results in time-dependent exposure when time is measured in gestational age. Not properly accounting for such time-dependent exposure can cause bias in the estimated exposure effect [2,3,4]. In other words, if one simply models vaccine exposure as yes or no, and ignores the different timings among the women who have received the vaccine, then the fact that a woman has received the vaccine late during pregnancy, instead of being indicative of her low risk of spontaneous abortion or preterm delivery independent of the vaccine, can be falsely attributed as the protective effect of the vaccine [4].

In addition, in studies on vaccine safety carried out using pregnancy exposure cohorts, women may also enter a study at any time during their pregnancy. An example of one ongoing prospective pregnancy exposure cohort study evaluating the safety of vaccines and medications is that conducted by the Organization of Teratology Information Specialists (OTIS) Collaborative Research group based at the University of California San Diego as part of the national Vaccines and Medication in Pregnancy Surveillance System (VAMPSS) [5,6]. For spontaneous abortion or preterm delivery, the fact that the pregnancies are not followed from the beginning (*i.e.*, gestational age zero, first day of the last menstrual period), causes downward bias in estimation of spontaneous abortion or preterm delivery rates [7]. This is because women who have had an early event of spontaneous abortion or, to a lesser extent, preterm delivery, tend not to be captured in the cohort study. Such data is said to be left truncated, and survival analysis methods need to be applied in order to properly estimate the event rates.

As the pregnancy safety studies are typically observational in nature, it is inevitable that potential confounders need to be taken into consideration. The Cox proportional hazards model can be used to handle left truncated spontaneous abortion or preterm delivery data, as well as to adjust for potential confounders in a regression setting. In addition, the Cox model can properly handle the time-dependent vaccine exposure caused by women receiving vaccine at arbitrary times during pregnancy.

There can be many potential confounders for spontaneous abortion or preterm delivery in association with vaccine exposure. Some examples are: maternal age, race/ethnicity, socioeconomic status, tobacco or alcohol use, pre-pregnancy body mass index, use of vitamin supplements, pregnancy history, previous preterm delivery, infection, fever, maternal asthma, depression, autoimmune disease, hypertension, and additional vaccine exposures [8,9,10,11]. Meanwhile these outcomes have relatively low observed incidence rates, which are typically at 10% or lower in the OTIS studies. This leads to relatively small number of events suggesting that propensity score can be a useful tool for simultaneously adjusting for multiple confounders.

Traditionally, when the exposure status is binary yes or no, the propensity score can be calculated using a logistic regression model with the confounders as predictors. With time-dependent vaccine exposure, while one can still calculate the propensity score using logistic regression by ignoring the timing of exposure and treating it as binary yes or no, one has the additional option to calculate it using the Cox model with time to exposure as the dependent variable. In the following we compare the two approaches using Monte Carlo simulation, as well as apply the Cox regression propensity score approach to a recent study of pandemic H1N1 vaccine exposure in pregnancy with time-dependent confounders.

## 2. Experimental Section

### 2.1. Data and the Model

Here, we consider preterm delivery, while the data and model for spontaneous abortion are similar. The time scale is gestational age, with the first day of a woman's last menstrual period (LMP) as time zero, and the day of preterm delivery or lost to follow-up (*i.e.*, last contact) as possibly right-censored event time. In addition, the day of enrollment is the left truncation time. In its most general form, the Cox model with time-dependent covariates can be written

(1) $$\lambda (t)=\lambda_{0}(t)\exp\{\beta^{\prime }Z(t)\}$$

where *λ*(*t*) is the hazard function for preterm delivery at time *t*, *λ*_0_(*t*) the baseline hazard, *β* is a vector of regression coefficients, and *Z*(*t*) is a vector of possibly time-dependent covariates. For example, in the case of vaccine exposure, denote *S* the time at which exposure occurs, then *Z*(*t*) = 0 for *t* < *S*, and *Z*(*t*) = 1 for *t* ≥ *S*. For women who are not exposed to the vaccine during their pregnancy, we can set *S* = ∞ so that effectively their *Z*(*t*) = 0 for all *t*. In addition, *Z*(*t*) can include time-independent covariates such as maternal race/ethnicity, that component of *Z*(*t*) being constant throughout time. Finally, if propensity score is used, then *Z*(*t*) includes propensity score as a component. See below for more details.

### 2.2. Simulation Setup

We carry out Monte Carlo simulation studies to investigate the two different approaches to calculate the propensity score to be included in Equation (1) in addition to the time-dependent vaccine exposure. To keep the simulation mechanism from getting too complex, we do not simulate left truncation in the data below; however, the approaches are intended to be used in pregnancy exposure cohort studies as will be illustrated later. In the following we simulate three confounders, a time-dependent exposure, and a right-censored event time. We consider two scenarios of how the exposure and its timing are generated.

In the first scenario, we have the following five steps:

*Step 1*: simulate the three confounders *X*_1_, *X*_2_ and *X*_3_ as independent standard normal N(0, 1).
*Step 2*: simulate an intermediate dichotomous exposure status *E* = 1 (exposed) or 0 (unexposed), given *X*_1_, *X*_2_ and *X*_3_ using a probit model:
  $$P(E=1|X_{1},X_{2},X_{3})=\Phi (\gamma_{0}+\gamma_{1}X_{1}+\gamma_{2}X_{2}+\gamma_{3}+X_{3})$$
  where Φ is the standard normal cumulative distribution function, and (*γ*_0_, *γ*_1_, *γ*_2_, *γ*_3_) = (1,1,1,1). Note that this is not the final exposure status; in fact *γ*_0_ = 1 tends to give more *E* = 1's than 0's at this point, but some of the *E* = 1's are never exposed following the steps below.
*Step 3*: among those with *E* = 1, generate the potential exposure time *S* given *X*_1_, *X*_2_ and *X*_3_ from an exponential distribution with rate exp(*α*_0_ + *α*_1_*X*_1_ + *α*_2_*X*_2_ + *α*_3_*X*_3_), where (*α*_0_, *α*_1_, *α*_2_, *α*_3_) = (1, 1, 1, 1). For those with *E* = 0 set *S* = ∞.
*Step 4*: generate the event time *T* given *X*_1_, *X*_2_, *X*_3_ and *Z*_1_(*t*) = *I*(*t* > *S*), where *I*(·) is the indicator function, according to a Cox model with hazard function
  (2) $$\lambda (t)=\lambda_{0}(t)\exp\left\{\beta_{1}Z_{1}(t)+\beta_{2}X_{1}+\beta_{3}X_{2}+\beta_{4}X_{3}\right\}$$
  where *λ*_0_(*t*) = 1, *β*_1_ takes on various values as given in the tables, and (*β*_2_, *β*_3_, *β*_4_) = (1, 1, 1). This is effectively a piecewise exponential distribution, with different rates on the two intervals (0, *S*) and (*S*, ∞). For more details of how to simulate under piecewise exponential distributions see [12].
*Step 5*: generate censoring time *C* as Uniform (0, *b*), where *b* is chosen so that a pre-specified percentage of censoring is achieved.

The final data set consists of the potentially right censored event time *X* = min(*T*, *C*), event indicator *δ* = *I*(*T* < *C*), covariates *X*_1_, *X*_2_, *X*_3_, and time-dependent exposure *Z*_1_(*t*) = *I*(*t* > *S*) for *t* ∈ (0, *X*). Note that if an observation has *E* = 1 in Step 2, and *S* > *X*, then *Z*_1_(*t*) = 0 on (0, *X*), and the individual is never exposed.

In the second scenario, we skip Step 2 above, and instead generate a potential exposure time *S* for every individual according to Step 3, followed by Steps 4 and 5. Once again, if *S* > *X* = min(*T*, *C*) then the individual is in fact unexposed.

The difference between the two scenarios above might be interpreted as follows. In the first scenario, some individuals inherently do not get vaccinated during pregnancy, while some other individuals end up not getting vaccinated because they have waited too long and the pregnancy has ended. In the second scenario, everyone has the potential to get vaccinated, some end up getting it during pregnancy, some end up not. The first scenario attempts to at least partially mimic the logistic regression set up, while the second scenario follows the Cox regression model. In practice we may not be able to tell which is the true data generating mechanism. The purpose of this investigation is to see if either of the propensity score methods might be sensitive to the different underlying assumptions.

Once the data have been generated, we build the propensity score using two approaches. The first approach uses logistic regression by treating the final exposure status *Z*_1_(*X*) as binary outcome, and with predictors *X*_1_, *X*_2_ and *X*_3_. Denote the estimated coefficients from this logistic regression as *ψ̂*_0_, *ψ̂*_1_, *ψ̂*_2_ and *ψ̂*_3_, then the linear combination PS_1_ = *ψ̂*_1_*X*_1_ + *ψ̂*_2_*X*_2_ + *ψ̂X*_3_ is a monotone function of the estimated probability of final exposure. We use PS_1_ as the propensity score, and fit the final adjusted Cox regression model for preterm delivery

(3) $$\lambda (t)=\lambda_{0}(t)\exp\{\beta_{1}Z_{1}(t)+\beta_{2}\;\cdot \;{\text{PS}}_{1}\}$$

In the second approach to build propensity score we use the Cox regression model by treating time to exposure as outcome, and with predictors *X*_1_, *X*_2_ and *X*_3_. For those never exposed in the data, time to exposure is censored at the end of the observation time. Denote the estimated coefficients from this Cox regression as *ϕ̂*_1_, *ϕ̂*_2_ and *ϕ̂*_3_, then the linear combination PS_2_ = *ϕ̂*_1_*X*_1_ + *ϕ̂*_2_*X*_2_ + *ϕ̂*_3_*X*_3_ is a monotone function of the estimated risk of exposure under the Cox model. We use PS_2_ as the propensity score, and fit the final adjusted Cox regression model

(4) $$\lambda (t)=\lambda_{0}(t)\exp\{\beta_{1}Z_{1}(t)+\beta_{2}\;\cdot \;{\text{PS}}_{2}\}$$

In the following we carry out simulation with sample size 100, and about 25% right-censoring. This gives about 75 events which is comparable to the vaccine data below.

## 3. Results and Discussion

### 3.1. Monte Carlo Simulation Results

Table 1 gives the simulation results under the first scenario, where an intermediate exposure status was first generated as binary in “Step 2”, then timing of exposure was generated for those “exposed”, as described in the last section. This “Step 2” generated about 70% exposed cases initially; but following Steps 3, 4 and 5 since *S* > *X* = min(*T*, *C*) makes an individual effectively unexposed during the whole observation period, we ended up with about 35% exposed cases. In the table we included estimates of *β*_1_ under the true Cox model (2), using the logistic propensity score approach (3), and using the Cox model propensity score approach (4). The table gives the average of the estimates over 2,000 simulation runs, its standard deviation (SD), the average of the estimated standard error (SE), the mean squared error (MSE), and the coverage probability (CP) of the nominal 95% confidence intervals. From the table we see that the true Cox model (2) does well for *β*_1_ = 0.5,1 or 1.5, as expected. The logistic propensity score approach has some bias when *β*_1_ = 0.5, with relatively low coverage probability of 89% and relatively high MSE compared to the other two approaches. But as *β*_1_ increases, the Cox model propensity score approach has increasing downward bias, leading to increasing MSE and coverage probabilities of 95% confidence intervals as low as 91%.

Table 2 gives the simulation results under the second scenario, where every subject has a potential exposure time. In contrast to Table 1, the logistic propensity score approach performed visibly poorly, with substantially larger MSE than the other two approaches, and coverage probability as low as 45%. From the table we also see large bias and substantial underestimation of the SD (by SE) in the logistic propensity score approach. On the other hand, the true Cox model performed well as expected, and the Cox model propensity score approach also performed very well. It should be acknowledged that in this scenario the Cox model is the true mechanism for generating the time-dependent exposure. In addition, compared to Table 1, the Cox propensity score model had on average about 63 exposure events to estimate the propensity score, while for Table 1, the Cox propensity score model had only about 35 exposure events. This might also explain the improved performance by the Cox model propensity score approach.

**Table 1:** First scenario of simulation: intermediate exposure status generated as binary, then timing of exposure is generated.

*β*_1_	**Model**	**Estimate**	**SD**	**SE**	**MSE**	**CP (%)**
0.5	True	0.511	0.269	0.267	0.073	95.3
	Logistic PS	0.700	0.253	0.262	0.104	89.2
	Cox PS	0.440	0.258	0.264	0.070	95.2

1	True	1.022	0.265	0.261	0.071	95.0
	Logistic PS	1.087	0.237	0.256	0.064	95.9
	Cox PS	0.909	0.264	0.257	0.078	93.1

1.5	True	1.535	0.265	0.261	0.071	95.2
	Logistic PS	1.480	0.237	0.253	0.057	96.5
	Cox PS	1.382	0.275	0.254	0.089	90.7

Notes: Sample size 100, about 25% right-censored. Proportion of exposed: about 35%. 2,000 simulation runs. “SD” = standard deviation, “SE” = standard error, “MSE” = mean squared error.

**Table 2:** Second scenario of simulation: every subject has a potential exposure time, some occurred after end of pregnancy.

*β*_1_	**Model**	**Estimate**	**SD**	**SE**	**MSE**	**CP (%)**
0.5	True	0.509	0.316	0.310	0.100	94.9
	Logistic PS	1.108	0.433	0.297	0.557	45.1
	Cox PS	0.505	0.313	0.310	0.098	95.0

1	True	1.022	0.311	0.301	0.097	94.5
	Logistic PS	1.434	0.376	0.295	0.329	63.9
	Cox PS	1.005	0.308	0.301	0.095	94.8

1.5	True	1.535	0.297	0.288	0.089	94.7
	Logistic PS	1.862	0.344	0.285	0.249	71.6
	Cox PS	1.506	0.294	0.287	0.086	95.0

Notes: Sample size 100, about 25% right-censored. Proportion of exposed: about 63%. 2,000 simulation runs. “SD” = standard deviation, “SE” = standard error, “MSE” = mean squared error.

Overall the logistic and the Cox model propensity score approaches appear to have comparable performances under the first simulation scenario. Note that under the first simulation scenario the Cox model does not reflect the true mechanism for generating the time-dependent exposure, and neither does the logistic regression model. But under the second simulation scenario, the results from the logistic propensity score approach appear unreliable. This is perhaps due to the fact that the logistic propensity score model does not adequately capture the data generating mechanism, whereas the Cox propensity score model does. Such sensitivity of the logistic propensity score approach to the underlying data generating mechanism makes it not suitable for general use in the presence of time-dependent exposure.

### 3.2. Analysis of Vaccine Data Using Time-dependent Propensity Score

A prospective cohort study of pandemic H1N1-vaccine (pH1N1)-exposed pregnancies and unexposed comparison pregnancies was carried out in order to assess the risks and relative safety of the pH1N1-containing vaccines during pregnancy [8]. Women residing in the U.S. or Canada were recruited during pregnancy and followed to outcome between October 2009 and August 2012. The primary outcomes included birth defects, spontaneous abortion, preterm delivery, and small for gestational age. The exposed group consisted of women exposed to either the monovalent (2009–2010 season) or trivalent (2010–2012 seasons) pH1N1-containing vaccine between LMP and the end of pregnancy. The unexposed group consisted of women who received no influenza vaccine of any type throughout pregnancy. Outcomes were collected by maternal interview and by medical records obtained from the obstetrician, pediatrician, and delivery hospital as well as pathology reports if relevant. As an illustration of the Cox model propensity score approach, here we focus on preterm delivery, which was defined as delivery at less than 37 completed gestational weeks.

**Figure 1:**
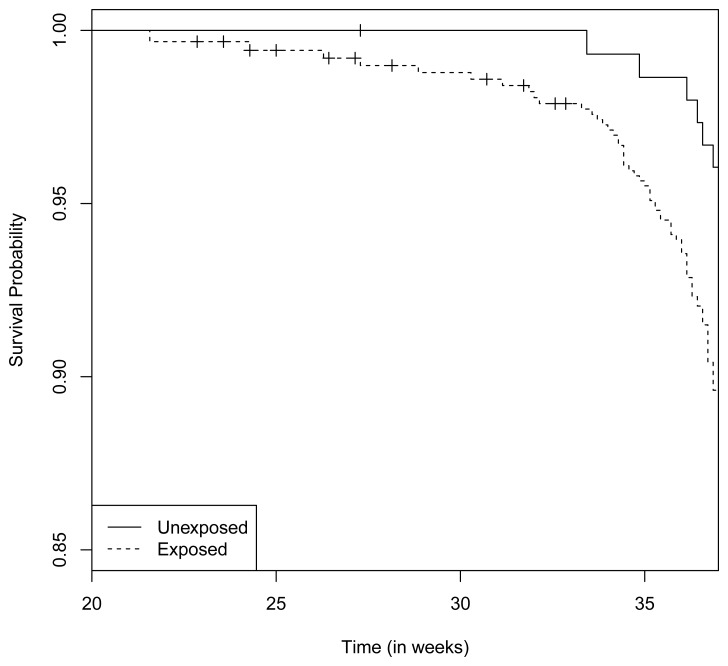
Left truncated Kaplan-Meier curves of one minus preterm delivery rates from the pH1N1 vaccine data.

**Figure 2:**
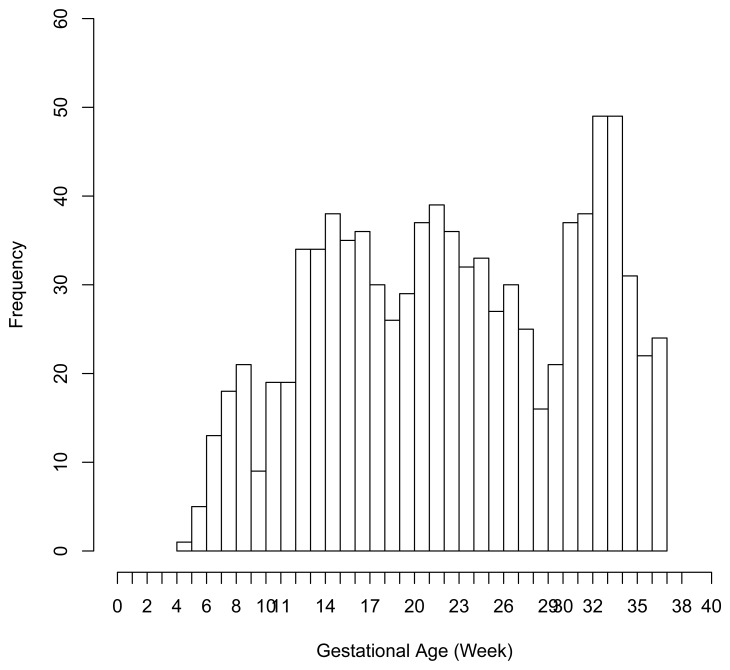
Enrollment (*i.e.*, left truncation) time in gestational age from the pH1N1 vaccine data.

**Figure 3:**
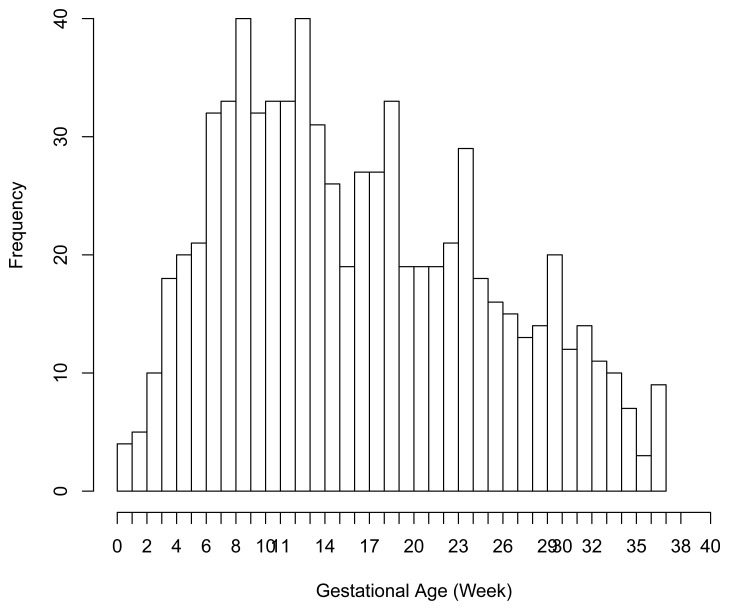
Vaccination time in gestational age from the pH1N1 vaccine data.

There were 6 preterm deliveries among 160 unexposed women, and 69 preterm deliveries among 753 exposed women (Figure 1), all enrolled before 37 weeks of completed gestation. There were 20 cases lost to follow-up after enrollment. Figure 2 shows the enrollment time in gestational weeks of all women, and Figure 3 shows the vaccination times. Follow-up started at enrollment for all women. Using the time-dependent covariate proportional hazards model (1), the unadjusted hazards ratio (HR) is 2.93 with 95% confidence interval (CI: 1.27, 6.75), indicating elevated risk for preterm delivery among exposed women. The time-independent confounders from [8] included maternal race/ethnicity (white, black, hispanic, or other), previous preterm delivery (yes/no), and autoimmune disease (yes/no). Due to the a *priori* inclusion and exclusion criteria for the study, autoimmune disease only occurred among the vaccine exposed women, so that it could not be balanced using propensity score. In addition, in the 2009–2010 season some women received the 2009–2010 seasonal vaccine (not containing the pH1N1 strain) prior to the pH1N1 monovalent vaccine becoming available, and were subsequently vaccinated with the monovalent pH1N1 vaccine. In this way, seasonal vaccine is a time-dependent confounder that also cannot be balanced using propensity score. On the other hand, influenza circulation season is a potential time-dependent confounder affecting every pregnant woman. Here we define the influenza season to be from October 1st of every year to March 31st of the next year, except for the 2009–2010 year we define it to be 1 April 2009, to 31 May 2010 [13]. Notice that while the vaccine exposures can only change from “no” to “yes” during a pregnancy, the time-dependent influenza season covariate can be “no” to “yes” to “no”, or “yes” to “no” to “yes” for some women. Finally, influenza infection before vaccination is also a potential time-dependent confounder. The details for coding time-dependent covariates were described in [3].

We build a propensity score for pH1N1 vaccine exposure consisting of maternal race/ethnicity, previous preterm delivery, influenza season and influenza infection, using the time-dependent covariate Cox model. Notice that in this case one cannot use the logistic regression to build a propensity score, since two of the confounders are time-dependent. From the Cox model fit we have

$$\begin{array}{c} \text{PS}(t)=-0.5968\times \text{race}.\text{black}+0.0670\times \text{race}.\text{hispanic}-0.4757\times \text{race}.\text{other}\\ +0.2910\times \text{previous}.\text{preterm}+2.2967\times \text{flu}.\text{season}(t)+0.1514\times \text{flu}.\text{infection}(t) \end{array}$$

Notice that this is a time-dependent propensity score. Using it together with autoimmune disease and seasonal vaccine exposure as covariates, the adjusted hazards ratio (HR) for preterm delivery associated with pH1N1 vaccine exposure becomes 2.46 with 95% CI (1.02, 5.93). In comparison, directly adjusting for the six covariates (race/ethnicity, previous preterm delivery, influenza season, influenza infection, autoimmune disease and seasonal vaccine exposure) gives an adjusted HR of 2.47 with 95% CI (1.02, 5.98). In this second adjusted model, pH1N1 vaccine exposure, previous preterm delivery and autoimmune disease are significant predictors of preterm delivery (*p*-value < 0.05), while black race/ethnicity is marginally significant (*p*-value = 0.054). In the first adjusted model with propensity score only pH1N1 vaccine exposure and autoimmune disease are significant predictors. The fact that the two adjusted HR's are basically identical appears to validate the Cox model propensity score approach, even though the true data generating mechanism in this case is unknown.

We note that the analysis here is slightly different from the preterm delivery analysis of Chambers *et al.* [8] in the following ways. Lost to follow-up subjects were included in this analysis as right-censored data while [8] included only pregnancies with known live born infants. Propensity score approach was not used in [8] for preterm delivery, nor was influenza season or influenza infection considered as potential confounders. The analysis here mainly serves as an illustration of the time-dependent propensity score approach using the Cox model.

## 4. Conclusions

In this paper we have compared the performance of two approaches to build propensity scores for time-dependent vaccine exposure during pregnancy: using logistic regression ignoring the timing of vaccine exposure, or using the Cox regression to model time to vaccine exposure. Our simulation results indicate that the Cox model approach should be preferred. In addition, the Cox model propensity score can also accommodate time-dependent confounders, such as the influenza season that we illustrated in the pH1N1 vaccine data analysis, to form a time-dependent propensity score. Several other time-dependent covariates may be relevant to vaccine safety in pregnancy studies, e.g., vaccines other than the one of primary interest, pregnancy induced hypertension/preeclampsia, time of entry into or access to prenatal care [14]. However, care must be taken to consider the complex relationships that may exist between factors that might affect propensity to be vaccinated, might confound the association between vaccination and preterm delivery, or may be in the causal pathway between vaccination and the outcome of preterm delivery.

Time-dependent propensity score was considered in Li *et al.* [15] and Lu [16], where its theoretical balancing property was established. To understand this, it helps to think of the time-dependent exposure as a stochastic process, also called counting process in the literature. This is a process that equals zero from time zero until the exposure occurs, then it jumps to one and stays there. In other words, it counts the number of exposure(s) for each individual, which is either zero or one; meanwhile it also incorporates the time dimension. In contrast, the traditional (time-independent) exposure is a binary random variable, without the time dimension. The time-dependent propensity score at time *t*, as modeled by the Cox proportional hazards model in this paper, reflects the probability of an individual becoming exposed at time *t*, given its history (including the covariates history) up until just before time *t*. This also contrasts the traditional propensity score, often modeled by the logistic regression model, as the probability of exposure given the observed covariates. The balancing property then says that given the propensity score at time *t*, the probability of an individual becoming exposed at time *t* given its history, no longer depends on the observed covariates.

Li *et al.* [15] and Lu [16] applied time-dependent propensity score to discrete exposure times in longitudinal studies, where the outcomes of interest are continuous. Here we have applied it to continuous exposure time, with continuous time-to-event outcomes that are subject to right-censoring (and left truncation). In our simulation and data analysis we used regression adjustment with the time-dependent propensity score. Alternatively one might consider matching or stratified analysis. In continuous time, matching or stratifying are more challenging, since with probability one there is at most one exposure occurring at any given time. One might further consider to discretizing the time, depending on the data at hand. In our case if we discretize the gestational age into discrete weeks, and we propose that preterm delivery can occur any time between 28 and up to 37 weeks of gestation, so that there are 10 discrete weeks. The remaining challenge is then the small number of events, with only 6 preterm deliveries in the unexposed group, making it insufficient for a matched or stratified analysis. With a different data set, however, matching or stratified analysis could well be viable alternatives to the regression adjustment using time-dependent propensity score.

Some clinicians have raised concerns over potentially different effects of different timing of vaccine in pregnancy. Chambers *et al.* [8] analyzed separately the effects of vaccination during the first, second, and third trimester, by comparing each of these vaccinated groups with the unexposed group. We did not repeat these analyses in this paper, since Section 3 mostly serves as an illustration of how the time-dependent propensity score might be used. The current approach can certainly be applied to such subgroup analysis. Different methodology, however, is needed if we are to examine the timing of vaccine in a more continuous fashion, and we are currently carrying out this research under a separate project.
